# Biodegradation of olive mill solid waste by *Anthracophyllum discolor* and *Stereum hirsutum*: effect of copper and manganese supplementation

**DOI:** 10.1186/s40643-025-00842-3

**Published:** 2025-03-10

**Authors:** V. Benavides, A. Serrano, F. Pinto-Ibieta, O. Rubilar, G. Ciudad

**Affiliations:** 1 Programa de Doctorado en Ciencias de Recursos Naturales. Facultad de Ingeniería y Ciencias, Universidad de La Frontera, Temuco, Chile; 2 Departamento de Ingeniería Química, Facultad de Ingeniería y Ciencias, Universidad de La Frontera, Temuco, Chile; 3 Instituto de Investigación del Agua, Universidad de Granada, 18071 Granada, Spain; 4 Escuela de Ciencias Ambientales y Sustentabilidad. Facultad de Ciencias de la Vida, Universidad Andres Bello, Santiago, Chile; 5 Departamento de Microbiología, Facultad de Farmacia, Universidad de Granada, Campus Universitario de Cartuja s n, Granada, Spain; 6 Departamento de Procesos Industriales, Facultad de Ingeniería, Universidad Católica de Temuco, Casilla 15-D, Temuco, Chile; 7 Centro de Excelencia en Investigación Biotecnología Aplicada al Ambiente (CIBAMA), Universidad de La Frontera, Temuco, Chile; 8 Instituto del Medio Ambiente (IMA), Universidad de La Frontera, Avenida Francisco Salazar 01145, Temuco, Chile

**Keywords:** Biological pretreatment, Inducers, Phenols, Lignin, White-rot fungi

## Abstract

*Anthracophyllum hirsutum* and *Stereum hirsutum* produce manganese peroxidase (MnP) and laccase to break down lignin, a potential biological pretreatment for lignocellulosic biomass. This work aimed to evaluate the effect of copper (Cu) and manganese (Mn) added to olive mill solid waste (OMSW) inoculated with *A. discolor* and *S. hirsutum* on ligninolytic enzyme activity, lignin degradation, and phenolic compound removal. Different optimal metal dosages were determined for each fungal strain. For *S. hirsutum*, the addition of 6.1 mg Cu kg⁻^1^ and 7.3 mg Mn kg⁻^1^ resulted in 173 ± 5 U·L⁻^1^ MnP activity (an 863% increase compared to no metal addition) and 42 ± 3% lignin degradation (183% higher than with no metal addition, 15 ± 3%). These conditions also led to 65–75% phenol removal efficiencies in OMSW at 25 days and 80–95% in leachates between 20 and 30 days. For *A. discolor*, 14.6 mg Mn kg⁻^1^ yielded 37 ± 7 U·L⁻^1^ MnP activity (a 142% increase compared to no metal addition) and 38 ± 7% lignin degradation (150% higher than with no metal addition, 15 ± 3%). Under these conditions, *A. discolor* achieved 80–90% phenol removal in leachates at 20 days. These results demonstrate the positive effect of optimised metal supplementation, highlighting the potential of *S. hirsutum* and *A. discolor* for effective lignocellulosic biomass pretreatment and future mycoremediation processes.

**Graphical Abstract:**

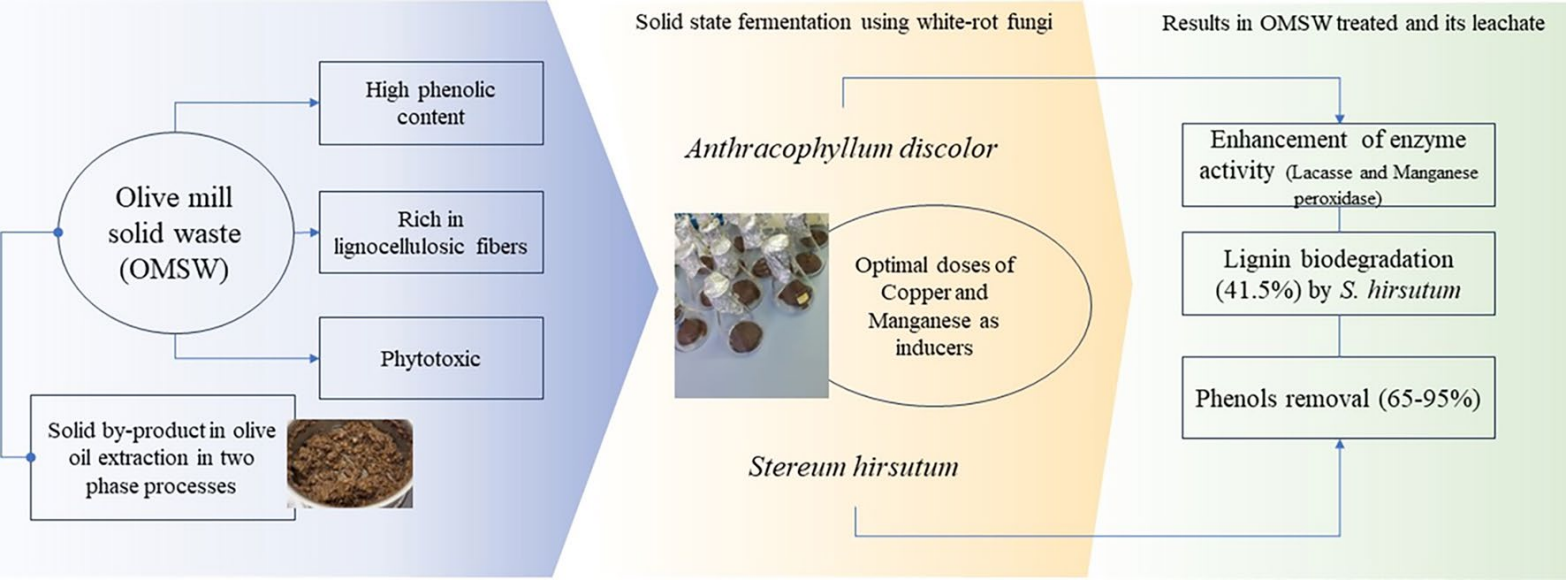

**Supplementary Information:**

The online version contains supplementary material available at 10.1186/s40643-025-00842-3.

## Introduction

Around 1000 Gt of lignocellulosic biomass, including wheat straw, sugarcane bagasse, and corn stalks, are generated annually (Al-Mallahi et al. 2016). The molecular structures of these biomasses are complex due to the presence of lignin, which forms a strong bond with cellulose and hemicellulose (Ning et al. 2021). Physicochemical treatments have been widely used to break the structure of the lignocellulosic biomass, improving their valorisation. Nowadays, the use of a wide variety of biocatalysts from white-rot fungi (WRF) in the form of crude enzymatic extracts enriched in laccase (Lac), manganese peroxidase (MnP), and manganese-independent peroxidase (MniP) is gaining attention as an alternative to physicochemical treatments (Chaudhary et al. 2023; Khalil 2023). The main advantages of fungal treatments include that they do not imply the use of toxic organic solvents (Sheldon and Woodley 2018), do not require a high energy consumption (El-Gendi et al. 2022), and, when the fungi are applied directly, a crude enzymatic extract can be easily obtained during the treatment for further application, like xenobiotic removal (Bilal and Iqbal 2020; Corbu et al. 2023).

Despite the potential interest in fungal treatments, their efficiency still needs to be enhanced. Several strategies can be implemented during WRF grown to improve this process, including the addition of metals as inducers and or cofactors (Baldrian 2003; Vrsanska and Langer 2016) and the nutrient balance through carefully selecting substrates (Atilano-Camino et al. 2020; Henske et al. 2018; Mishra et al. 2017) or choosing an appropriate fungal strain (Tortella et al. 2005). Adding a wide variety of inducers and cofactors, such as Cu, Mn or Fe, can improve the ligninolytic enzymatic activity in WRF (Acevedo et al. 2011; Baldrian 2003; Hermosilla et al. 2017; Reddy and Kanwal 2022; Rubilar et al. 2011). Inducers serve as mediators to assist the bioconversion of several compounds, and their presence can increase the kinetics of reactions by enhancing the enzymatic activity (da Silva Vilar et al. 2022). Cofactors act on the transcriptional level, and they are part of the architecture of enzymes with a wide variety of functions that mainly involve electron transfer, O_2_ binding, activation, and reduction, NO_2_^–^ and N_2_O reduction, and substrate activation (Sekretaryova et al. 2019).

The effect of metal addition has been usually evaluated based on the added doses. For example, Cu addition of 0.25 mM in the culture medium can increase the laccase activity by *Ganoderma lucidum* up to eight times compared to culture media without this metal (Kuhar and Papinutti 2014). Mn dosage has been reported to promote MnP from *A. discolor* and *Fomes sclerodermeus* in a wide range from 0.2 to 2.0 mM (Acevedo et al. 2011). However, it is worth noting that the microbial response could be more related to bioavailable metal fractions or, if the substrates contain a notable concentration of metals, the total metal concentration in the medium (Pinto-ibieta et al. 2016).

On the other hand, lignocellulosic fibers in agricultural biomasses can promote the production of ligninolytic enzymes during fermentation processes, thereby boosting WRF growth and enzymatic extract potential (Atilano-Camino et al. 2020). Andriani & Tachibana (2016) demonstrated that ligninolytic enzyme production (laccase and lignin peroxidase) from *Bjerkandera adusta* SM46 depends on the type of lignocellulosic material used, achieving the highest laccase and manganese peroxidase activities (1.1 and 0.4 U g, respectively) using rice straw in comparison with kapok fibers, wood meal, or pulp waste. Benavides et al. (2022) evaluated the ligninolytic enzyme activity of *A. discolor* and *S. hirsutum* using OMSW as substrate. The OMSW acted as a growth support and substrate to the fungi and promoted the secretion of extracellular ligninolytic enzymes, with both WRF strains reaching a high OMSW biodegradability. Also, these authors stated that using local strains would be beneficial since they are already adapted to the environmental conditions and would not be subjected to special regulations as would be a foreign organism.

Therefore, applying both strategies, metal addition and the use of the lignocellulosic substrate, could result in synergistic effects for enhancing the enzymatic activity and obtaining a high-value product, an enzymatic extract, for further biotechnological applications (Atilano-Camino et al. 2020). Therefore, this work aimed to assess the increase in the enzymatic activity from WRF through (1) Cu and Mn addition in liquid-state fermentation by *A. discolor* in culture medium, and (2) a combination of Cu and Mn addition and OMSW as substrate in solid-state fermentation (SSF) by *A. discolor* and *S. hirsutum,* separately. The processes were evaluated according to enzyme production (Lac, MnP, and MniP), lignin degradation, and phenol removal.

## Materials and methods

### Microorganisms

The present research used *A. discolor* Sp4 and *S. hirsutum* as fungal strains. The selection, isolation and storage of the fungi was previously described in Benavides et al. (2022) (more details are also provided in supplementary material). Before the experiment phases, the strains were activated in potato dextrose agar medium (39 g of PDA in 1 L of distilled water and sterilised in an autoclave for 20 min at 121 °C). From that, a 6 mm disk of fungi was used as initial inoculum for the assays, obtained after an incubation for 7 days at 28 °C (Benavides et al. 2022).

### Olive Mill Solid Waste (OMSW)

Olive mill solid waste (OMSW), used as a substrate for fungi growth, was collected from the olive mill “Olivares de Quepu” in Pencahue, Chile. Prior to use, the OMSW was stored at − 20 °C to avoid undesirable fermentation processes.

### Effect of copper and manganese on enzymatic activity by *A. discolor* in liquid medium

The effect of copper and manganese on ligninolytic enzyme production was evaluated by using various concentrations from stock solutions of CuSO_4_·5 H_2_O and MnSO_4_·H_2_O in a medium with glucose (15 g L^−1^), peptone from potatoes (5 g L^−1^) and yeast extract (2.5 g L^−1^) (pH 5.5). The assessed concentrations reported of Cu and Mn were selected following previous research (Table 1) (Acevedo et al. 2011; Vrsanska and Langer 2016). 100 mL of culture medium in 250 mL Erlenmeyer flasks were inoculated with five 6 mm agar disks of *A. discolor* (details described in 2.1) and incubated for 30 days at 28 °C. Each condition was assessed in triplicate.

**Table 1:** Conditions for evaluation of the effect of inducer cofactors on enzymatic activity from *Anthracophyllum discolor*

Trial No	Cu (mg L^−1^)	Mn (mg L^−1^)
1	0.0	0.0
2	25.5	0.0
3	0.0	72.8
4	25.5	72.8
5	30.7	36.4
6	12.7	87.8
7	12.7	36.4

### Solid-state fermentation

For inoculum preparation, a 250 mL Erlenmeyer flask containing 100 mL of culture medium with glucose, peptone from potatoes, and yeast extract at pH 5.5 (described in Sect. 2.3) was autoclaved at 121 °C for 20 min. Each flask was initially inoculated with five 6 mm agar disks of active mycelia (Sect. 2.1) and then incubated for 14 days at 28 °C. Finally, a liquid inoculum was obtained by blending the previous fungal cultures for 1 min.

For the solid-state fermentation (SSF) assay, 10 g of sterilised OMSW at 121 °C for 20 min in Erlenmeyer flasks (100 mL) were used, adjusting moisture to 80%. The effect of two cofactors on enzymatic extracts production was tested (Table 2), copper sulfate (CuSO_4_·5 H_2_O, Sigma-Aldrich) in different concentrations (0.0–1.0 mM) according to Vrsanska et al. (2016) and concentrations of monohydrated manganese sulfate (MnSO_4_·H_2_O, Sigma-Aldrich), to 0.00–2.0 mM, were used in this assay according to Acevedo et al., (2011). After cooling, the flasks were autoclaved and inoculated with 1 mL of blended fungal mycelia (Rubilar et al. 2011). Negative controls were included as flasks without fungal inoculum. The SSF experiments were run for 30 days in the dark at 28 °C. The effect was evaluated using a response-surface methodology (RSM) of central composite design (CCD).

**Table 2:** Culture conditions for olive mill solid waste treatment in solid-state fermentation using *Anthracophyllum discolor and Stereum hirsutum*, where total metal concentration is the sum of the metal concentration initially contained in the substrate and the added metals

Trial No	Metal added		Total metal content	
	Cu (mg kg^−1^)	Mn (mg kg^−1^)	Cu (mg kg^−1^)	Mn (mg kg^−1^)
1	0.0	0.0	8.48	13.0
2	5.1	0.0	13.58	13.0
3	0.0	14.6	8.48	27.56
4	5.1	14.6	13.58	27.56
5	6.1	7.3	14.62	20.28
6	2.5	17.6	11.02	30.56
7	2.5	7.3	11.02	20.28

### Analytical methods

Each analytical determination was carried out at least in triplicate. Previously to the experiment, the total content of Cu and Mn in OMSW was analysed from 0.5 g of sample digested with 10 mL of HNO_3_ 5% (Microwave Digestion of US EPA 3051-Solid Sample) and measured by inductively coupled plasma—mass spectrometry (EPA Method 6020B -SW-846) (United States Environmental Protection Agency 2014). Extract recovery and enzymatic activity quantification were performed using the spectrophotometric methodology described by Hermosilla et al. (2017). More details about the determination of Lac, MnP and MniP can be found in Benavides et al. (2022). Total phenolic content was quantified by spectrophotometry with a gallic acid (GA) calibration curve using the Folin-Ciocalteu method. The results were expressed as mg GAE L^−1^ for the leachate and as mg GAE 100 g^−1^ of OMSW for samples obtained in SSF. More details about the extraction process before the phenolic quantification can be found in the supplementary material. The phenolic profile was determined by a Hitachi Primaide high-performance liquid chromatograph coupled to a diode array detector (HPLC–DAD) equipped with a Kromasil^®^ C18 column (more details in supplementary material). Lignin content in the OMSW was quantified as acid-insoluble Klason lignin (TAPPI T222-om02) and acid-soluble lignin (TAPPI UM 250). The difference between the lignin content before and after the fungal treatment was used to calculate the percentage of lignin degradation according to Hermosilla et al. (2018) (more details in supplementary material).

## Results and discussion

### Effect of Cu and Mn addition on enzymatic activity by *A. discolor* in liquid state fermentation

**Fig. 1:**
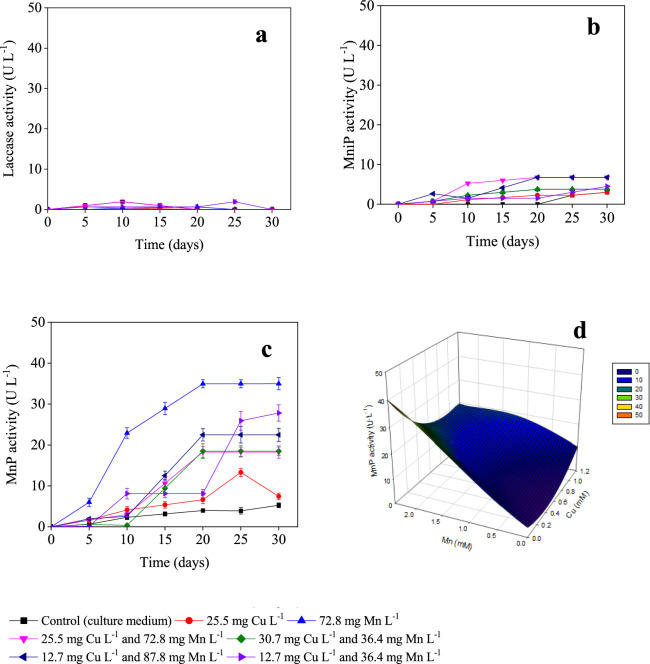
Effect of Mn and Cu on laccase (**A**), manganese-independent peroxidase (**B**), and manganese peroxidase activity (**C**) produced by *Anthracophyllum discolor* in liquid medium, and response surface plot of manganese peroxidase activity at 20-day (**D**)

The variations of Lac, MnP, and MniP were monitored about the different metal doses using *A. discolor* in liquid-state fermentation using a culture medium as substrate (Fig. 1). MnP was the enzyme that reached a higher production than Lac and MniP, with concentrations below 2 U L^−1^ and 8 U L^−1^, respectively. Similar results were reported by Hermosilla et al. (2017) using WRF with metal addition as Fe and Mn in a substrate with wheat straw and Kirk’s media, where the enzymatic activity of MniP was under 5 U L^−1^. The highest production of MnP was detected at 20 days of treatment for a dose of 2 mM Mn, reaching a value of 35 ± 1 U L^−1^ (Fig. 1A). This value was around 9 times higher than the control (no metal addition), i.e., 4 ± 0.3 U L^−1^ at the same 20 days. The positive effect of Mn dosage on MnP activity in *A. discolor* has been previously reported by Acevedo et al. (2011), where adding 0.25 mM of Mn showed the best results, achieving 1354 U L^−1^ of MnP activity.

The response surface plot for the values obtained at 20 days showed a positive effect of Mn dose on MnP activity when the total concentration of this metal increased from 20.4 to 30.6 mg L^−1^ (Fig. 1B). This is possible because MnP is a heme-containing enzyme consisting of Mn-binding sites, and it catalyses the oxidation of Mn^2+^ to Mn^3+^ in the presence of H_2_O_2_ (Kannaiyan et al. 2015). Thus, Mn^2+^ plays a key role in the transcriptional phase and the activation of the peroxidase enzyme (Baldrian 2003; Slavens et al. 2019). On the other hand, adding Cu slightly favoured the enzyme activity at increasing Cu concentrations, although to a more limited extent than Mn (Fig. 1B). This increase could be associated with Cu having an important role in regulating peroxidase activity, causing induction or suppression of the enzyme activity depending on the concentration of nutrients (Slavens et al. 2019).

The combined addition of Cu and Mn negatively affected the MnP activity compared to the individual addition of the metals (Fig. 1B). Concretely, MnP activity decreased up to 50% compared to the individual addition of Mn, but it was better than when only copper was added (Fig. 1B). These results were consistent with the values obtained on day 20, where the MnP activity in the treatments with both metals in different concentrations was in the range of 7 ± 0.3 U L^−1^ to 22 ± 1 U L^−1^ (Fig. 1A), three times higher than the control, but 17% lower than the optimum seen for the Mn dose of 2.41 mM (35 ± 1 U L^⁻^ of MnP). These results may be associated with (1) a high concentration of metals in the extracellular environment that limits the development of the ligninolytic enzyme system (Baldrian 2003); (2) under this condition, metal excess, probably copper, can also cause oxidative damage of proteins by the induction of oxidative stress associated with the production of reactive oxygen species like hydroxyl or superoxide radicals (Stohs and Bagchi 1995), while Mn accumulation can be generating consequences on the cellular level (vacuoles for storage and degradation and Mn export), restricting the associated process to Mn (Robinson et al. 2021); and (3) enzymes are not protected by cell-associated metal-detoxication mechanisms (Baldrian 2003).

### Effect of Cu and Mn addition on enzymatic activity by *A. discolor* and *S. hirsutum* in solid-state fermentation

#### A. discolor

**Fig. 2:**
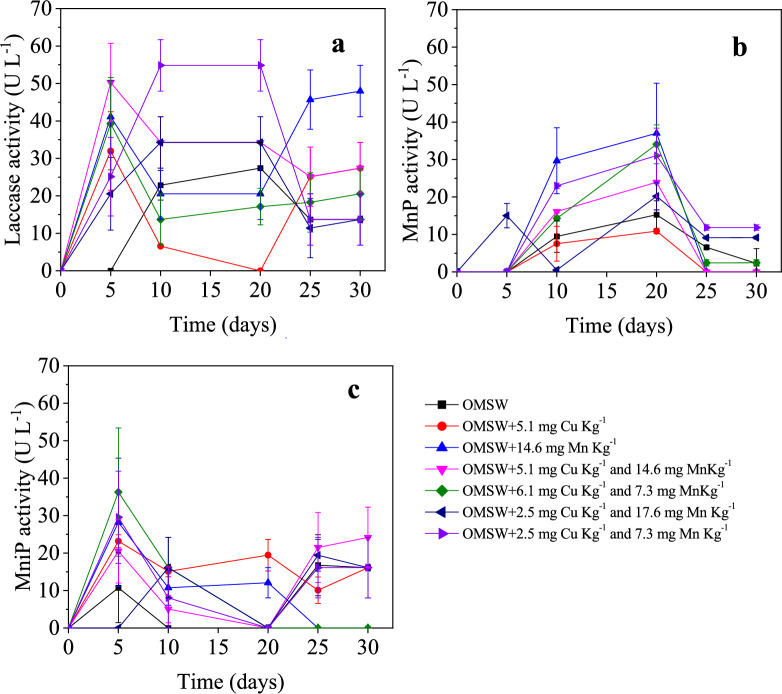
Laccase (**A**), manganese peroxidase (**B**), and manganese-independent peroxidase activity variation (**C**) as a function of different doses of copper and manganese for *Anthracophyllum discolor* in solid-state fermentation using olive mill solid waste (OMSW) as substrate

Lac was the main ligninolytic enzyme produced by *A. discolor*. The highest Lac activity was determined for the combined dose added of 7.3 mg Mn kg^−1^ and 2.5 mg Cu kg^−1^, reaching a mean value of 55 ± 7 U L^−1^ (137 ± 17 U kg_OMSW_^−1^) from days 10 to 20 (Fig. 2A). This value was 2.2 times higher than the determined for the control (no metal addition). However, metal doses of 17.6 mg Mn kg^−1^ and 2.5 mg Cu kg^−1^ decrease the Lac activity by around 50% compared to the above-mentioned optimal activity. Similarly, individual Cu addition strongly affected the Lac activity at days 10–20, i.e., 90–100% reduction compared to the optimal value. Therefore, these results explain that there is also an optimal dosage of metals that, added together and in appropriate concentrations, may be an alternative to increasing the lac activity from *A. discolor*. However, adding these compounds to an exceeding concentration can result in an inhibition of enzyme production.

The observed positive effects of Cu in combination with Mn, but its inhibitory behaviour when added alone concerning the control, was related by Kannaiyan et al. (2015) to cross-regulation phenomena, reporting that Mn can act as an inducer to Lac activity. That would explain why the improvement in the enzymatic activity was higher by adding combined doses than by adding individual metals. Likewise, the limited response in the Lac activity to the Cu dose would be because of a lack of MnP activity by the absence of Mn supplementation. A minimal MnP activity would be required to release the phenols contained in the lignocellulosic fibers and, thus, promote the Lac activity closely related to phenol degradation (Gianfreda et al. 1999; Mehra et al. 2018).

Despite the adverse effects of some metal doses compared to the control, the results were markedly higher than the Lac values determined in the previous liquid-state fermentation assays, i.e., less than 2 U L^−1^ (Fig. 1A). Similarly, the results of MnP activity in the control at days 10 and 20 were higher than those obtained with liquid-state culture media (Sect. 3.1) in the same period, increasing 5 U L^−1^ at day 10 and 3 U L^−1^ at day 20 (Fig. 1B). These enhancements may be associated with the use of a complex matrix as substrate, with high lignocellulosic contents (35 ± 3% cellulose, 33 ± 4% lignin, and 45 ± 8% of hemicellulose) that could act as enzyme inducer, favouring the secretion of both enzymes by the fungi (Andriani and Tachibana 2016; Benavides et al. 2022; Kheirkhah et al. 2020) (Andriani and Tachibana 2016; Benavides et al. 2022; Kheirkhah et al. 2020).

The highest value of MnP activity in solid-state fermentation with *A. discolor* was determined for a metal dose of 14.6 mg of manganese kg^−1^, reaching a value of 37 ± 13 U L^−1^ (92 ± 33 U kg_OMSW_^−1^) at day 20 (Fig. 2B). This value was more than 2 times higher than the determined for the control, which reached a maximum MnP activity of 153 ± 3 U L^−1^ (38 ± 9 U kg_OMSW_^−1^) on day 20. The individual dose of Cu did not show any difference in the MnP activity compared to the control without metal dose. The combined doses of Cu and Mn increased the MnP activity compared to the control, where the dose of 2.5 mg Kg^−1^ of Cu and 7.3 mg kg^−1^ of Mn showed the best results within combined doses, reaching a value of 31 ± 7 U L^−1^ (78 ± 18 U kg_OMSW_^−1^). This value was similar to the obtained for the individual dose of 14.6 mg kg^−1^ of manganese (Fig. 2B). The positive effect of the Mn dose was expected since this metal has been shown to play a key role in the transcriptional phase as well as in the regulation of either laccase or peroxidase activity, causing either induction or suppression of the enzyme activity, respectively, depending on the nutrient concentration (Slavens et al. 2019). Mn directly participates in lignin degradation in white-rot fungi through the reaction cycle of Mn-dependent peroxidase (MnP) (Baldrian 2003).

A positive effect was also observed for the MniP at the different metal doses compared to the control without metal addition (Fig. 2C). This improvement could be due to the enhancement observed for the Lac and MnP activities, which release organic compounds initially comprised of the lignocellulosic structures and phenols. As a result, these compounds become more bioavailable for fungi biodegradation, which activates more enzymatic mechanisms, including MniP (Giardina et al. 2000; Petruccioli et al. 2009).

**Fig. 3:**
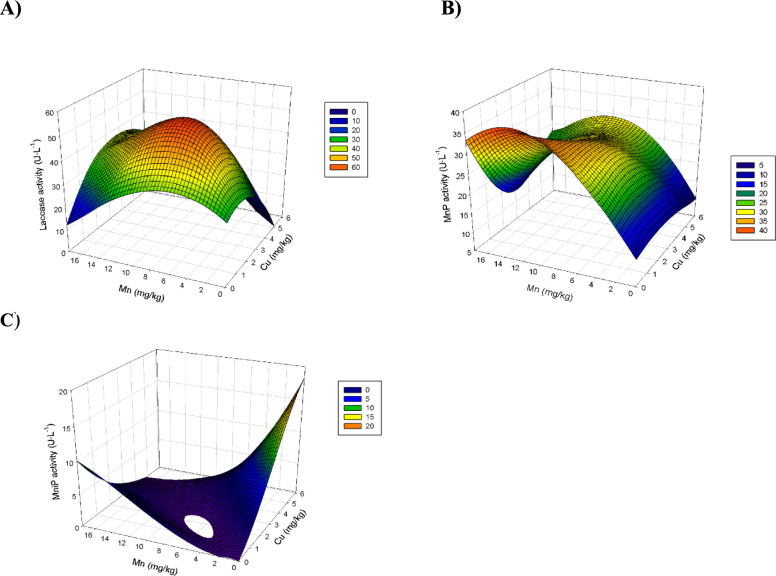
Three-dimensional surface plots of manganese peroxidase activity produced by *Anthracophyllum discolor* at 20-day in solid-state fermentation using olive mill solid waste (OMSW) as substrate. Plot of Lac activity as a function of copper and manganese (**A**); Plot of MnP activity as a function of copper and manganese (**B**); Plot of MniP activity as a function of copper and manganese (**C**)

The response surface plot for Lac, MnP, and MniP activities at day 20 by *A. discolor* is shown in Fig. 3. Lac activity showed optimal values at intermediate doses of Mn and Cu, i.e., between 8.0 and 14 mg kg^−1^ of Mn and 2.0–4.0 mg kg^−1^ of Cu. Increasing the individual metal amounts decreased the Lac activity (Fig. 3A). This improvement in Lac activity obtained with individual doses was enhanced even when the metals were added together in the same concentration ranges, reaching activity values above 40 U L^−1^, around 10 U L^−1^ higher than that predicted for the control. The individual effect of manganese on MnP activity was favourable at increasing concentrations (Fig. 3B); however, the MnP activity was not influenced by the individual addition of Cu at the assessed range (Fig. 3B). The combined dose of Cu and Mn can improve the MnP activity at doses below 14.0 mg kg^−1^ of Mn. However, at doses higher than 14.0 mg kg^−1^ of Mn, the co-addition of even low concentrations of Cu, i.e., 1–3 mg kg^−1^, resulted in a marked decrease in the MnP activity (Fig. 3B). The individual doses of metals enhanced the MniP activity compared to the control, while the combined effect was negative in every case (Fig. 3C). This trend should be carefully considered because these results correspond to the values obtained at 20 days, which was considered optimal for all the enzymatic activities. However, MniP activity might be different from the process behaviour. In fact, according to Fig. 1C, positive effects were observed in the combined metal doses in the other sampling days. Given the results, perhaps the optimal values to improve the enzymatic activity of the three types of enzymes would be the addition of medium concentrations of Mn (6–12 mg kg^−1^) and low concentrations of Cu (1–2 mg kg^−1^).

#### S. hirsutum

**Fig. 4:**
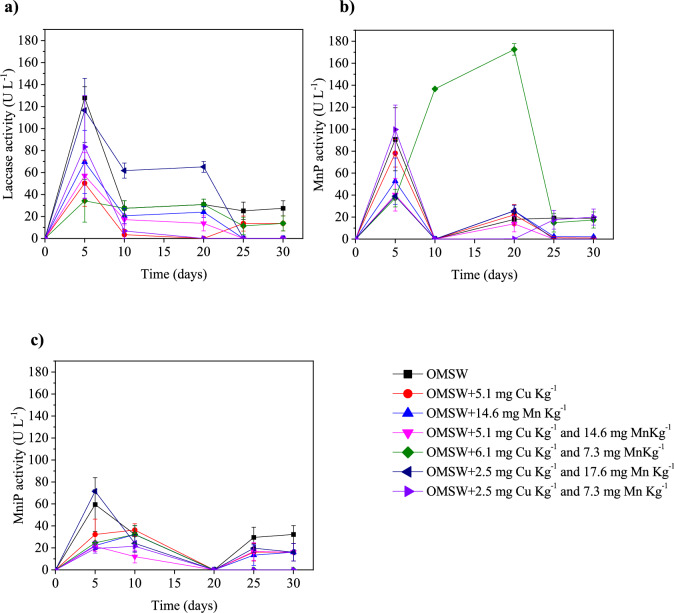
Laccase (**A**), manganese peroxidase (**B**), and manganese-independent peroxidase activity variation (**C**) as a function of different doses of copper and manganese for *Stereum hirsutum* in solid-state fermentation using olive mill solid waste (OMSW) as substrate

The evolution of enzymatic activities determined for each metal dose to *S. hirsutum* using OMSW as substrate shows that the highest Lac activity was determined on day 5 at the control, obtaining 128 ± 10 U L^−1^ (320 ± 25 U kg_OMSW_^−1^) (Fig. 4A). This peak in the Lac activity rapidly decreased to values below 30 U L^−1^, i.e., around 79% lower than at day 5. However, the metal dose of 2.5 mg kg^−1^ of Cu and 17.6 mg kg^−1^ of Mn maintained the Lac activity at values higher than 60 U L^−1^ (around 150 U kg_OMSW_^−1^) up to day 20 (Fig. 4A). The metal concentration in the OMSW was enough to result in higher Lac activity in the control than at the other evaluated metal doses. The highest individual dose of Cu, i.e., 5.1 mg Cu kg^−1^, strongly affected the Lac activity, showing almost negligible values at most of the experimental times. This inhibition caused by excessive copper may be linked to the direct toxicity of free copper ions and the production of a toxic compound, which could result in oxidative stress at an advanced stage of fungal growth and be responsible for limited transcriptional induction (Palmieri et al. 2000). The alteration of the target protein to inhibit metal binding, metal-restricted uptake to prevent cellular interference, and overproduction of targeted proteins to control or compensate for the loss of functions directly related to the level toxicity of metal are some other resistance mechanisms developed by fungi under conditions of copper excess. These strategies may ultimately have an impact on the secondary metabolism of the fungus (Robinson et al. 2021).

The highest values of MnP activity by far were determined for a metal dose of 6.1 mg kg^−1^ of Cu and 7.3 mg kg^−1^ of Mn, reaching a value of 137 ± 1 U L^−1^ (342 ± 2 U kg_OMSW_^−1^) at day 10 and 173 ± 5 U L^−1^ (431 ± 13 U kg_OMSW_^−1^) at day 20 (Fig. 4B). These values were 9.6 times higher than the determined for the control, which reached a maximum MnP activity of 18 ± 5 U L^−1^ (45 ± 13 U kg_OMSW_^−1^) on day 20. The individual dose of the metal also resulted in a positive effect compared to the control at day 20, reaching a value of 22 ± 9 U L^−1^ (55 ± 9 U kg_OMSW_^−1^) with the addition of 5.1 mg kg^−1^ of Cu and 26 ± 5 U L^−1^ (64 ± 13 U kg_OMSW_^−1^) with the addition of 14.6 mg kg^−1^ of Mn, i.e., around 25% compared to the control with no metal addition (Fig. 4B). These values are higher than the results obtained for *A. discolor* in the same experimental period in comparison to the control (without Cu or Mn), showing that the improvements depend on other parameters despite the dosed metal concentration, such as the requirement of the used strains and/or the metals provided by the substrate itself (Liu et al. 2020).

For MniP activity, no significant differences were observed with the addition of metals compared to the control, showing a range of values between 0 and 72 ± 1 U L^−1^ (Fig. 4C). MniP activity produced by *S. hirsutum* was similar to those obtained in the treatments under solid-state fermentation with *A. discolor* (Fig. 2C), where almost all treatments were values under 40 U L^−1^. However, *S. hirsutum* allowed the increase of enzymatic activity after day 20 by at least 70% compared to the results obtained under liquid fermentation conditions using commercial media, which were always under 7 U L^−1^ (Fig. 1B). This suggests that using lignocellulosic waste as a growth substrate and supplementary inducer is a valid strategy to enhance enzymatic extract production (Merino et al. 2019; Slavens et al. 2019).

**Fig. 5:**
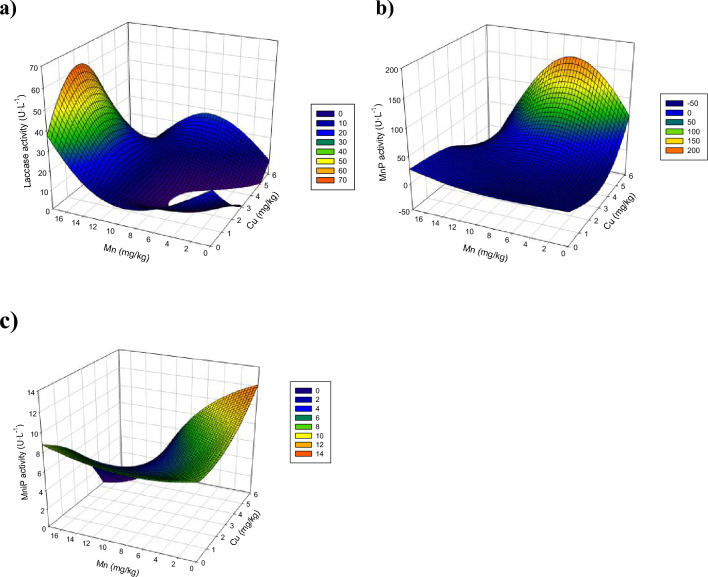
Three-dimensional surface plots of enzyme activity produced by *Stereum hirsutum* at 20-day in solid-state fermentation using olive mill solid waste (OMSW) as substrate. Plot of Lac activity as a function of Mn and Cu (**A**); Plot of MnP activity as a function of Mn and Cu (**B**); Plot of MniP activity as a function of Mn and Cu (**C**)

The response surface plots for Lac, MnP, and MniP activities at day 20 by *S. hirsutum* are shown in Fig. 5. The Lac activity showed the highest values at the combination of the highest Mn concentrations and intermediate Cu concentrations (Fig. 5A). However, MnP activity was favoured at high Cu and intermediate Mn concentrations. The Cu dose strongly influenced MnP activity, the values of which were almost constant at Cu concentrations below 4 mg kg^−1^ regardless of the dosed Mn (Fig. 5B). The individual dose of Cu favoured MniP activity. However, the individual dose of Mn did not substantially impact the MniP activity. Furthermore, MniP activity was negatively affected by increasing the concentration of both metals when dosed together. Therefore, according to the response surface plots, it is not possible to determine an optimal dose concentration of both metals for all the enzyme activities, showing the contradictory requirements Lac and MnP activities. That would imply that the metal dose strategy needs to be adjusted to the concrete enzyme activity, the enhancement of which is more desirable at each time.

### Effect of Cu and Mn on lignin degradation by *A. discolor* and *S. hirsutum* in solid-state fermentation

**Fig. 6:**
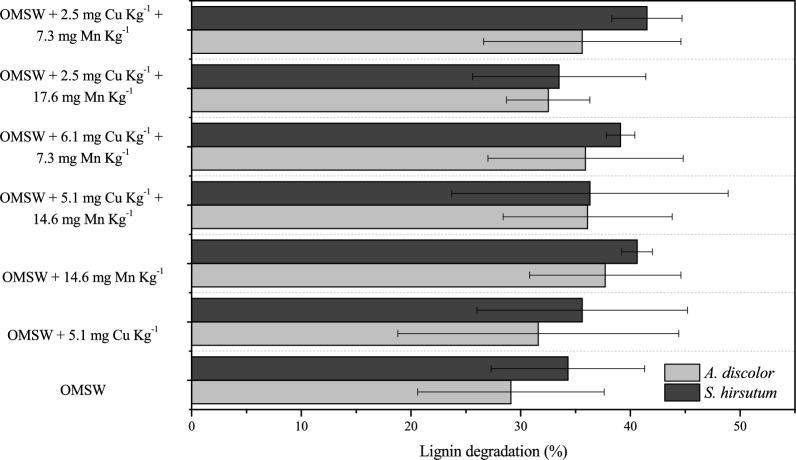
Lignin degradation (%) by *Anthracophyllum discolor* and *Stereum hirsutum* in solid-state fermentation with different metal doses at day 20

The highest level of lignin degradation, 42 ± 3% at 20 days, was obtained with the lowest dose of Cu (2.5 mg kg^−1^) and intermediate dose of Mn (7.3 mg kg^−1^) in the treatment with *S. hirsutum* (Fig. 6). For *A. discolor*, the highest level of lignin degradation (38 ± 7%) after 20 days was obtained with the maximum individual Mn dose (14.6 mg kg^−1^). The positive effect associated with both strains of Mn dose can directly result from the improvement of the MnP activity described in Sect. 3.2. Moreover, Sari et al. (2016) reported that improving the MnP activity favours the oxidising of Mn^2+^ to Mn^3+^ by the MnP. The highly reactive Mn^3+^ can combine with organic chelating compounds and acts as a low-molecular-weight, diffusible redox mediator targeting the oxidation of the phenolic components of lignin and, thus, enhancing the lignin degradation (Kannaiyan et al. 2015).

### Effect of Cu and Mn on phenolic compounds degradation from OMSW using *A. discolor* and *S. hirsutum* in solid-state fermentation

**Fig. 7:**
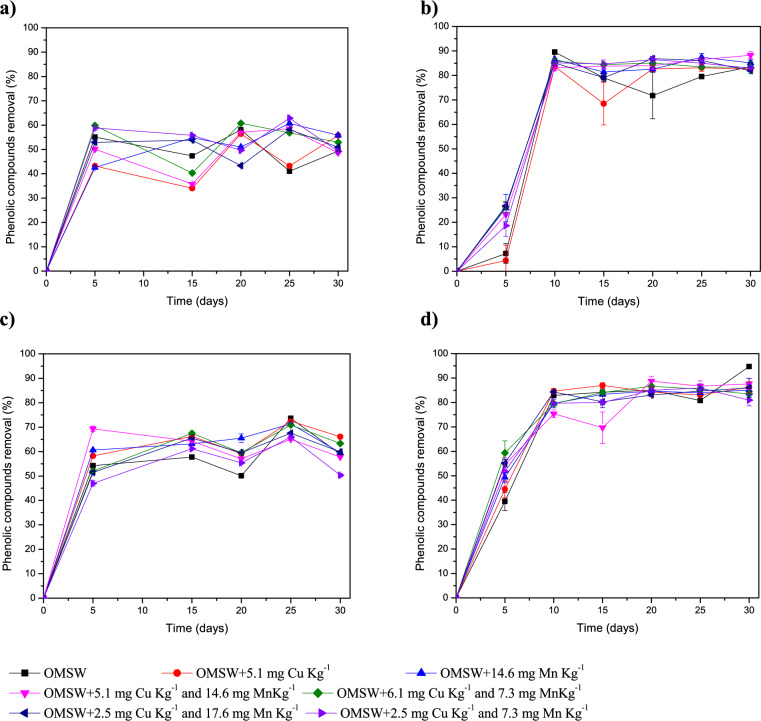
Phenol removal as a function of culture time using olive mill solid waste (OMSW) as substrate supplemented with metals for both strains studied: Removal in OMSW (**A**) and Removal in leachate by *Anthracophyllum discolor* (**B**)*;* Removal in OMSW (**C**) and Removal in leachate by *Stereum hirsutum* (**D**)

The effect of copper and manganese on OMSW treatment by *A. discolor* and S*. hirsutum* and the behaviour of phenolic compounds was evaluated by monitoring the total phenolic compound removal percentage throughout the experimental period in the OMSW (Fig. 7A, B) and its leachate (Fig. 7C, D). As noted, in most cases, no significant differences were found in the treatments with different individual and combined metal doses. *A. discolor* removed phenolic compounds effectively from OMSW, reaching stable values of 45–60% at 5 days (Fig. 7A), while leachate phenol removal values were 85–95% at 10 days (Fig. 7B). *S. hirsutum* achieved a stable removal efficiency of 65–75% of phenols in OMSW at 25 days (Fig. 7C), while in its leachate, the highest removal values were between 80 and 95% at 30 days. However, at 20 days, it is possible to observe an 80–90% phenol removal (Fig. 7D). Therefore, both strains could be good candidates for future mycoremediation processes for OMSW thanks to their oxidative ligninolytic system, which allows the lignin degradation and opening of phenyl rings (Benavides et al. 2022; Naraian 2019). The phenol removal efficiencies achieved in this work were close to those reported by other authors, such as Reina et al. (2013), who described an efficiency in phenol compound removal from OMSW of 90% by *Auricularia auricula-judae*, 80% by *B. adusta* and 100% by *Coprinellus radians*, all at 14-day under SSF without the addition of metals.

Table 3 shows the individual phenolic compound concentrations of the raw OMSW and at the OMSW treated with *A. discolor* and *S. hirsutum* and their leachates at day 20 when 2.5 mg kg^−1^ of Cu and 17.6 mg kg^−1^ of Mn was added. As can be seen, the final concentration of the measured individual phenolic compounds indicated that most of them were widely metabolized during the process (Table 3), consistent with the high total phenol removal efficiency described in Fig. 7. Despite most of the phenolic compounds being effectively degraded, some specific compounds were accumulated compared to the initial concentration during the fungal treatments. Specifically, in OMSW treated by *S. hirsutum* and its leachate*,* gallic acid hexoside, protocatechuic acid hexoside, and 3-hydroxytyrosol were detected (Table 3). In OMSW treated by *A. discolor* and its leachate, several compounds were accumulated concerning the raw OMSW, highlighting the accumulation of gallic acid hexoside, protocatechuic acid hexoside, 3-hydroxytyrosol, 3,4-dimethoxybenzyl alcohol, and 2,6-dimethoxyphenol (Table 3). The accumulation of these compounds would be related to the breakdown of the lignocellulosic fibers of the OMSW, entailing the release of the phenolic compounds integrated into these fibers (Luo et al. 2021). Likewise, some specific phenolic compounds can accumulate because they are intermediates of the incomplete degradation of other phenolic compounds (Serrano et al. 2017). The presence of a higher concentration of specific phenolic compounds in the OMSW treated by *A. discolor,* and its leachate is due to the lower Lac activity observed with this strain compared to *S. hirsutum* (Figs. 2A and 4A), which did not fully degrade these phenolic compounds.

**Table 3:** Individual phenolic compounds concentration (mg L^−1^) in olive mill solid waste (OMSW) and its leachate obtained from the solid-state fermentation assays using *Anthracophyllum discolor and Stereum hirsutum* at day 20 when 2.5 mg kg^−1^ of Cu and 17.6 mg kg^−1^ of Mn were added

	Untreated OMSW	Leachate from OMSW pretreated by *A. discolor*	Leachate from OMSW pretreated by *S. hirsutum*	OMSW pretreated by *A. discolor*	OMSW pretreated by *S. hirsutum*
Phenolic compounds					
4-Hydroxybenzoic acid	0.48 ± 0.038	ND	ND	ND	ND
Gallic acid hexoside	0.75 ± 0.06	1.02 ± 0.04	0.81 ± 0.01	ND	ND
3,4-dihydroxyphenylglycol	4.59 ± 0.39	ND	ND	ND	ND
Protocatechuic acid hexoside	ND	4.23 ± 0.28	3.36 ± 0.16	ND	4.26 ± 1.61
3-hydroxytyrosol	2.14 ± 0.01	1.93 ± 0.10	2.12 ± 0.05	ND	ND
p-tyrosol	0.16 ± 0.01	ND	ND	ND	ND
Vanillic acid	0.32 ± 0.01	ND	ND	ND	ND
3,4-dimethoxybenzyl alcohol	6.35 ± 0.10	1.37 ± 2.37	ND	6.76 ± 1.93	ND
Vanillin	11.0 ± 0.2	ND	ND	ND	ND
2,6-dimethoxyphenol	0.43 ± 0.01	16.76 ± 1.22	ND	5.42 ± 1.47	ND
Hydroxycinnamic acids and derivatives					
Coutaric acid	ND	0.76 ± 0.06	0.79 ± 0.04	Traces	Traces
Chlorogenic acid	1.27 ± 0.02	ND	ND	ND	ND
p-Coumaric acid	Traces	ND	ND	ND	ND
Trans-ferulic acid	0.10 ± 0.01	ND	ND	ND	ND
Quercetin 3-rutinoside	0.38 ± 0.02	ND	ND	ND	ND
Quercetin 3-glucoside	0.62 ± 0.01	ND	ND	ND	ND
Myricetin	0.23 ± 0.04	ND	ND	ND	ND
Quercetin	2.63 ± 0.02	ND	ND	ND	ND
5-Hydroxymethylfurfural	0.35 ± 0.01	1.90 ± 0.14	1.84 ± 0.11	Traces	0.23 ± 0.03

*ND* not detected

## Conclusions

Both fungal strains treated the OMSW under SSF conditions. Lac activity improvement was more related to Cu addition, whereas Mn addition improved MnP activity. The positive effect of Mn on MnP may have released phenols from the OMSW, explaining the positive relationship between Mn and Lac. The use of Cu and Mn enhanced specific enzymes, enabling the design of tailored supplements for specific fungal applications. This study demonstrated that Cu and Mn supplementation significantly improved enzymatic activities concerning the control with no metal addition. For *S. hirsutum*, MnP activity resulted in an 863% increase compared to the control, whereas lignin degradation was 183% higher than the control. For *A. discolor*, MnP activity increased by 142% and lignin degradation increased by 150%, respectively, compared to the control. These results demonstrate the positive effect of optimised metal supplementation, highlighting the potential of *S. hirsutum* and *A. discolor* for effective lignocellulosic biomass pretreatment and future mycoremediation processes. Future studies should prioritise scaling up these processes to pilot or demonstration levels, evaluating if these fungal strains can operate in real conditions, competing with other environmental microorganisms.

## Supplementary Information

Supplementary material 1.

## Data Availability

Not applicable.
