# Supplementary material for: Biodegradation of olive mill solid waste by Anthracophyllum discolor and Stereum hirsutum: effect of copper and manganese supplementation
Source: Bioresour Bioprocess. 2025 Mar 10;12(1):18. doi: 10.1186/s40643-025-00842-3 (PMC11893923; doi:10.1186/s40643-025-00842-3)
Supplement: Supplementary file 1 — Supplementary material 1. [file 40643_2025_842_MOESM1_ESM.docx]

**Biodegradation of olive mill solid waste by *Anthracophyllum discolor* and *Stereum hirsutum*: Effect of copper and manganese supplementation**

V. Benavides ^a,b,c^, A. Serrano ^c,d^, F. Pinto-Ibieta ^e^, O. Rubilar ^b,f,g^, and G. Ciudad ^b,f,g,*^.

^a^ Programa de Doctorado en Ciencias de Recursos Naturales. Facultad de Ingeniería y Ciencias, Universidad de La Frontera, Temuco, Chile.

^b^ Departamento de Ingeniería Química, Facultad de Ingeniería y Ciencias, Universidad de La Frontera, Temuco, Chile.

^c^ Instituto de Investigación del Agua, Universidad de Granada, Granada, 18071, Spain.

^d^ Departamento de Microbiología, Facultad de Farmacia, Campus Universitario de Cartuja s n, Universidad de Granada, Granada, Spain.

^e^ Departamento de Procesos Industriales, Facultad de Ingeniería, Universidad Católica de Temuco, Casilla 15-D, Temuco, Chile.

^f^ Centro de Excelencia en Investigación Biotecnologica aplicada al Ambiente (CIBAMA), Universidad de La Frontera, Temuco, Chile.

^g^ Instituto del Medio Ambiente (IMA), Universidad de La Frontera, Avenida Francisco Salazar 01145, Temuco, Chile.

* Corresponding author: Instituto del Medio Ambiente (IMA), Universidad de La Frontera, Avenida Francisco Salazar 01145, Temuco, Chile.

E-mail address: [gustavo.ciudad@ufrontera.cl](mailto:gustavo.ciudad@ufrontera.cl) (G. Ciudad).

**SUPPLEMENTARY MATERIAL**

**2. Materials and methods**

*2.1 Microorganisms*

The fungal strains *A. discolor* Sp4 and *S. hirsutum* were isolated from decayed wood in the temperate forests of southern Chile belonging to the Culture Collection of the Environmental Nanobiotechnology Laboratory at the Universidad de La Frontera, Chile (Benavides et al., 2023). The fungi were stored at 4 °C in Petri dishes with potato dextrose agar (PDA) medium until use. Then, the strains were activated in PDA medium (39 g of PDA in 1 L of distilled water and sterilized in an autoclave for 20 min at 121 °C) using one disk of 6 mm as the initial inoculum and incubated at 28 °C for 7 days for the assays (Benavides et al., 2023).

*2.5 Analytical methods*

*Total phenolic compounds*

Total phenol content was quantified by spectrophotometry with a gallic acid (GA) calibration curve, using the Folin-Ciocalteu method, expressing the results as mg GAE L^-1^ to leachate and mg GAE 100 g^-1^ of OMSW after treatment in SSF. Previous extractions were carried out for this determination using 5 g of OMSW and 5 mL of methanol-water (80:20 v/v). The mixture was stirred for 1 min in a vortex apparatus and centrifuged at 1200*g* for 10 min, and the methanol phase was separated. Five extraction cycles were performed for each sample, and the total volume obtained was mixed and used for analysis (García et al., 2016). For phenolic profile determination, samples were treated with a water-methanol-formic acid solution (24:25:1) and sonicated. They were left to stand for 24 h to sonicate, centrifuged, and filtered for further analysis. A Hitachi Primaide high-performance liquid chromatograph was used, coupled to a diode array detector (HPLC-DAD) equipped with a Kromasil® C18 column. The mobile phase was Milli-Q water acidified with 1% formic acid (A) and acetonitrile (B), with a flux of 1mL min^-1^ and the injection volume used was 10 µL.

*Lignin content*

The lignin content of OMSW was determined as acid-insoluble Klason lignin (TAPPI T222-om02) and acid-soluble lignin (TAPPI UM 250), according to the methodology described by Dence and Lin (1992). The percentage of lignin degradation produced by fungi was calculated as the difference between initial lignin content in untreated OMSW and lignin content in fungal-treated OMSW adjusted for weight loss according to Eq. (1) as per Hermosilla et al. (2018):

$Lignin content \left( \% \right)=\frac{\left( 1-W\left( L_{f} \right) \right)}{\left( W_{0}\left( L_{0} \right) \right)}x100$ Eq. (1)

where,

L_0_: lignin content in untreated OMSW (%)

L_f_: lignin content in OMSW after fungal treatment (%)

W: dry weight of OMSW after fungal treatment (g)

W_0_: dry weight of OMSW untreated (g)

**References**

Benavides, V., Pinto-Ibieta, F., Serrano, A., Rubilar, O., & Ciudad, G. (2022). Use of Anthracophyllum Discolor and Stereum Hirsutum as a Suitable Strategy for Delignification and Phenolic Removal of Olive Mill Solid Waste. Foods, 11(11). https: doi.org 10.3390 foods11111587

García, A., Rodríguez-Juan, E., Rodríguez-Gutiérrez, G., Rios, J.J., Fernández-Bolaños, J. (2016). Extraction of phenolic compounds from virgin olive oil by deep eutectic solvents (DESs). Food Chemistry, 197, 554-561.

Hermosilla, E., Rubilar, O., Schalchli, H., da Silva, A. S., Ferreira-Leitao, V., & Diez, M. C. (2018). Sequential white-rot and brown-rot fungal pretreatment of wheat straw as a promising alternative for complementary mild treatments. *Waste Management*, *79*, 240–250. https: doi.org 10.1016 j.wasman.2018.07.044
